# Pelvic insufficiency fractures in cervical cancer patients treated with definitive chemoradiation: impact of dosimetric and clinical factors

**DOI:** 10.1007/s00066-026-02518-z

**Published:** 2026-05-21

**Authors:** Rosa Autorino, Raffaella Michela Rinaldi, Davide Cusumano, Luca Russo, Benedetta Gui, Viola De Luca, Roberta Giannini, Maura Campitelli, Valentina Lancellotta, Gabriella Macchia, Nicolò Bizzarri, Maria Antonietta Gambacorta

**Affiliations:** 1 UOC Radioterapia Oncologica, Fondazione Policlinico Universitario Agostino Gemelli IRCCS, Largo Francesco Vito 1, 00168 Roma, Italy; 2 UO Fisica Medica e Radioprotezione, Mater Olbia Hospital, Olbia, Italy; 3 UOC Radiodiagnostica, Fondazione Policlinico Universitario “A. Gemelli” IRCCS, Roma, Italy; 4 UOC Radioterapia Oncologica, Responsible Research Hospital, Molise ART, Campobasso, Italy; 5 Woman, Child and Public Health Department, Fondazione Policlinico Universitario “A. Gemelli” IRCCS, Roma, Italy

**Keywords:** Bone marrow dosimetry, Radiation dose constraints, Osteoporosis risk, Pelvic radiotherapy toxicity, Age-related risk factors, External beam radiation therapy

## Abstract

**Background:**

The aim of this study was to investigate the correlation between clinical and dosimetric factors and the incidence of pelvic insufficiency fractures (PIFs) in patients with cervical cancer treated with radical radiotherapy (RT), with or without chemotherapy, and to identify dose constraints that may reduce this risk.

**Methodology:**

We reviewed data from cervical cancer patients treated with pelvic external-beam RT at our institution between 2020 and 2023. For each patient, pelvic bone marrow (BM) was contoured and divided into three subsites: lumbosacral spine (LSBM), ilium (IBM), and lower pelvis (LPBM). Data on the volume of each region receiving 10, 20, 30, and 40 Gy (V10, V20, V30, and V40, respectively) and D_mean_ were collected.

**Results:**

A total of 202 patients were retrospectively analyzed. Of these, 36 patients (17.8%) developed PIFs in the treatment field, with most cases being asymptomatic. Our analysis indicates that V40 of the LPBM is the most significant dosimetric parameter. When combined with patient age, this parameter stratifies the risk of fracture development according to the V40 value, thereby accounting for the patient’s age in the risk assessment.

**Conclusion:**

This preliminary study allowed us to identify the dose constraints to reduce the risk of PIF according to patient age. Further investigations are needed to confirm these findings by including these parameters in the planning process.

## Introduction

External beam radiotherapy combined with concomitant platin-based chemotherapy and followed by brachytherapy is widely considered the gold standard treatment for locally advanced cervical cancer. Moreover, recently introduced techniques, such as volumetric modulated arc therapy (VMAT), have led to optimization of the dose delivered to target lesions while reducing the risk to healthy tissue [1].

Due to the decrease in incidence and mortality rates of locally advanced cervical carcinoma over the past few years, significant attention has been focused on the long-term effects of treatments on the pelvis [2]. Pelvic insufficiency fractures (PIFs) represent a relatively common late toxicity after pelvic RT. These fractures usually occur when physiological loads are applied to previously weakened bones with decreased elastic resistance [3]. Various authors suggest that RT, often combined with other techniques to treat pelvic malignancies, could contribute to an increased risk of PIF development by damaging the bone matrix and reducing the vascular supply [4]. Furthermore, recent studies have underlined that women with cervical carcer who had received pelvic irradiation faced an increased risk of PIFs in contrast to those who did not undergo this treatment [5].

Despite the growing interest in late toxicity following pelvic RT, the incidence of PIFs remains relatively uncertain, ranging between 10% and 29% [6]. Various studies suggest that any condition that weakens the pelvic bones, such as osteoporosis, can be considered a potential risk factor for the development of pelvic fractures [7]. Therefore, postmenopausal status, lower body mass index (BMI), older age, and low bone mineral density before RT are recognized as potential contributing clinical factors increasing future fracture risk. A study by Waldenstrom et al. [8] also suggested an association between the mean dose absorbed by the pelvis and the onset of fractures and pain in the hip, sacrum, and pubic bones. However, there is still no definitive information regarding clinical and dosimetric parameters that contribute to damaging the pelvic bones.

Also, only few studies have addressed the association between dose–volume histogram (DVH) parameters and the risk of PIF development in patients treated with VMAT for locally advanced cervical carcinoma.

The aim of this study was to evaluate the impact of clinical and dosimetric parameters on the occurrence of PIFs after VMAT-RT combined with platin-based chemotherapy for locally advanced cervical cancer. Furthermore, this study aimed to identify possible dose constraints for the pelvis to prevent the onset of these fractures.

## Materials and methods

Medical charts of 202 adult female patients with a diagnosis of locally advanced cervical carcinoma who were treated at the Department of Radiation Oncology of Policlinico Gemelli in Rome from February 2019 to December 2023 were retrospectively collected. Only patients treated with definitive RT were enrolled in this study; patients undergoing surgical treatment and receiving postoperative adjuvant RT were excluded from the analysis.

All patients underwent exclusive radiochemotherapy treatment consisting of definitive external beam radiotherapy (EBRT) with VMAT, combined with concomitant weekly platin-based chemotherapy and followed by high-dose-rate (HDR) intracavitary brachytherapy (ICBT).

Other clinical characteristics were obtained from patient medical records and included the following:

- BMI
- Smoking status
- Diabetes
- Postmenopausal status
- Hormone replacement therapy (HRT)
- Documented pre-existing history of osteoporosis
- Vitamin D levels
- Concomitant steroid therapy

### Radiotherapy treatment

All patients received EBRT of the pelvis with VMAT, which offered better optimization of the dose delivered to target lesions while reducing the risk of potential side effects on the organs at risk (OAR), such as the bladder, small intestine, rectum, and femoral heads.

According to international delineation guidelines [9], the tumoral clinical target volume (CTV) contained the whole cervix, the uterus, the vagina, and parametrial tissue. The elective nodal target contained the obturator lymph nodes, the internal and external iliac lymph nodes, and the common iliac nodes. If the common iliac nodes were involved at the initial staging, the lumbo-aortic nodes were also included in the CTV. If the lower third of the vagina was involved at the initial staging (stage IIIA), the RT fields were extended to include the inguinal lymph nodes in the irradiated volume.

The planning target volume (PTV) was defined as the CTV +0.8 cm.

The total prescribed EBRT dose to the PTV was 45 Gy at 1.8 Gy per fraction. The VMAT plans were delivered using 10- or 15-MV photon beams. In the case of lymph node involvement, an additional dose of RT was delivered using the simultaneous integrated boost (SIB) technique, for a total of 55 Gy at 2.2 Gy for positive pelvic lymph nodes, or 57.5 Gy at 2.3 Gy for positive common iliac or para-aortic nodes.

The EBRT was followed by an additional brachytherapy boost to the cervical region, which was delivered with an HDR technique and consisted of a total dose of 28 Gy in 4 fractions of 7 Gy weekly to the high-risk CTV (HR-CTV).

The concurrent chemotherapy protocol consisted of weekly cisplatin at 40 mg/mq for a total of 5–6 weeks. If contraindicated, the concurrent chemotherapy regimen consisted of carboplatin AUC2.

In addition to the delineation of the common pelvic organs, pelvic bones were also delineated on the treatment planning CT scan.

The pelvic bone marrow (BM) was retrospectively delineated on the planning CT scan contouring all bones within the pelvis and further divided into three distinct subsites, including the lumbosacral spine (LSBM), ilium (IBM), and lower pelvis (LPBM), as described by Mell et al. [10]:

- LSBM: from the highest vertebral body included in the PTV (usually L4) to the entire sacrum
- IBM: from the iliac crests extending to the superior border of the femoral heads
- LPBM: extending from the superior border of the femoral heads to the inferior border of the ischial tuberosities, encompassing the pubes, ischia, acetabula, and proximal femora

The following parameters were recorded for the BM and each subsite: the mean dose and the volume of each region that received at least 10, 20, 30, and 40 Gy (defined as V10, V20, V30, and V40, respectively).

### Follow-up and PIF diagnosis

After the end of treatment, patients were enrolled in a follow-up program, which consisted of diagnostic tests including magnetic resonance imaging (MRI) to evaluate their response to therapy. Pelvic MRI was performed according to the following timeline: 3 months after the end of overall treatment and then every 3 months for the first 2 years.

Follow-up scans were retrospectively reviewed by radiologists and then compared with the diagnostic MRI to identify the possible onset of fractures in the pelvic region. All PIFs were encountered in the presence of bone marrow edema, characterized by low signal intensity (SI) on T1-weighted imaging (WI) and high SI on T2-WI and low-b-value diffusion weighted imaging (DWI), and/or in the presence of a fracture line, which appeared as a hypointense rim on both T1- and T2-WI [11]. The occurrence of PIFs, their number, and their location were noted. The onset of PIF was registered as the date of detection on MRI scans. In patients with multiple injuries detected, the date of the first event was recorded.

### Statistical analysis

A comprehensive database including dosimetric data, clinical characteristics, and information regarding the number, the location, and the onset of pelvic fractures was created. Subsequently, clinical and dosimetric parameters were analyzed in relation to the occurrence or absence of pelvic fractures, which was treated as a dichotomous outcome.

All patients were considered a training set due to the limited number of observed events. Univariate analysis was conducted using either the Wilcoxon–Mann–Whitney test or the *t* test, depending on the normality of data distribution, which was previously evaluated using the Shapiro–Wilk test [12]. A linear logistic regression model, which combined the two most significant variables, was created.

The predictive performance of the model was evaluated using the area under the receiver operating characteristic (ROC) curve (AUC), with 95% confidence intervals. The optimal cut-off threshold was determined by maximizing the Youden index (J), and sensitivity and specificity values at the best threshold were computed accordingly. The risk of pelvic fracture occurrence was then defined combining the two most significant variables, i.e., patient age (divided into five categories) and V40 of the LPBM.

The probability of the onset of a fracture was calculated using the following formula:
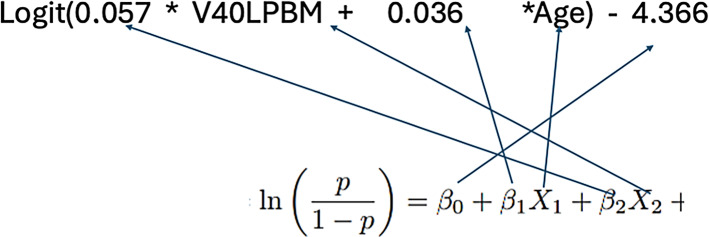


## Results

A total of 202 adult female patients were retrospectively included in this study. The median age was 54 years (range: 28–86 years). At the time of the diagnosis, 85 patients (42%) were in postmenopausal state. Eight postmenopausal patients were on HRT before undergoing RT treatment. All patients enrolled in this study were diagnosed with locally advanced cervical disease, defined according to the FIGO (2018) international staging system. Although the majority of patients had a pathological stage ranging from IIB to IIIC2, a few patients had other tumor stages (from stage IB2 to IVB).

The majority of patients (197 patients, 96.5%) received concurrent radiochemotherapy followed by brachytherapy.

Almost all patients received concomitant platin-based chemotherapy (96.5%); the majority (80%) received five cycles of cisplatin. Only 3.5% of patients (5/202) did not undergo this treatment.

Regarding brachytherapy treatment, the median minimal dose to 90% of the high-risk clinical target volume (D90) was 90 Gy (range: 85–95 Gy), with a median dose per fraction of 7.02 Gy (corresponding to 9.96 Gy EQD2, assuming an α/β of 10). Patients had an overall treatment time of 52 days (range: 50–56 days) and 100% compliance with the interventional radiotherapy (IRT).

Osteoporosis status was only available for 21 patients, 12 of whom had documented osteoporosis at diagnosis. Smoking status was reported for 36 patients, 35 of whom were active smokers at the time of clinical data collection. Patient, tumor, and treatment characteristics are summarized in Table 1.

**Table 1:** Patient, tumor, and treatment characteristics

Study population	*n* = 202
**Median age, year (range)**	**54 (28–86)**
**Median BMI, kg/**m^**2**^**(range)**	**24.19 (14.65–39.04)**
*FIGO stage (2018), n (%)*	
IB1	1 (0.5%)
IB2	3 (1.5%)
IIA1	5 (2.5%)
IIA2	4 (2%)
IIB	40 (20%)
IIIA	6 (3%)
IIIB	4 (2%)
IIIC1	82 (40.5%)
IIIC2	37 (18%)
IVA	16 (8%)
IVB	4 (2%)
*Concurrent chemotherapy*	
Yes	197 (96.5%)
No	5 (3.5%)
*Osteoporosis status*	
Yes	21 (10%)
No	181 (90%)
*Smoking status*	
Yes	36 (18%)
No	166 (82%)

In our study, 36 patients developed at least one pelvic fracture in the irradiated field, corresponding to an overall incidence of 17.8%. A total of 51 fractures were diagnosed on MRI. Overall, 12 patients (33%) developed symptoms at the time of MRI, such as pelvic or lumbar pain, while the rest of the patients remained asymptomatic.

The median time interval between the end of EBRT and the detection of PIF on MRI was 7 months (range: 1–24 months post treatment), in particular 14 PIFs occurred within 6 months, 16 within 12 months, 2 within 18 months, and 4 within 24 months following treatment.

According to the results of previous studies [13], most fractures were situated in the sacrum. Furthermore, 81% of patients developed at least one fracture in the sacrum. Other fracture sites included the bilateral sacral alae, the acetabulum, and the lumbar spine. Moreover, eight patients developed multiple PIFs during follow-up, with an average number of fracture sites of 2.

The following distribution of PIFs was observed: The lumbar vertebrae were fractured in only one patient, the sacrum was affected in 29 patients (81%), and the sacroiliac joint was involved in four patients (11%). Four patients developed fractures of the iliac alae, while the acetabulum was the site of fracture in four patients; finally, two patients had involvement of the pubic branches.

We evaluated the correlation between the incidence of PIFs and both clinical and dosimetric parameters through univariate analysis. In line with previous studies [7, 14, 15], age was identified as the most relevant predisposing clinical factor associated with the occurrence of PIFs. Other clinical features (such as diabetes, BMI, smoking), which have been reported to be associated with an increased fracture risk, did not show a statistically significant correlation with pelvic fractures in our series. Disease-related factors (such as tumor stage) were not assessed in this study. Although concomitant chemotherapy can increase the toxicity of RT, no significant impact on PIFs was observed in our analysis.

The dose delivered to the BM and each subsite was obtained through the corresponding DVHs. We evaluated the possible association between post-RT pelvic fractures and dosimetric parameters, such as mean dose, V10, V20, V30, and V40 of the BM and each subsite. Our data showed that V40 of the LPBM represents a significant predisposing parameter for the occurrence of post-radiation pelvic fractures.

**Fig. 1:**
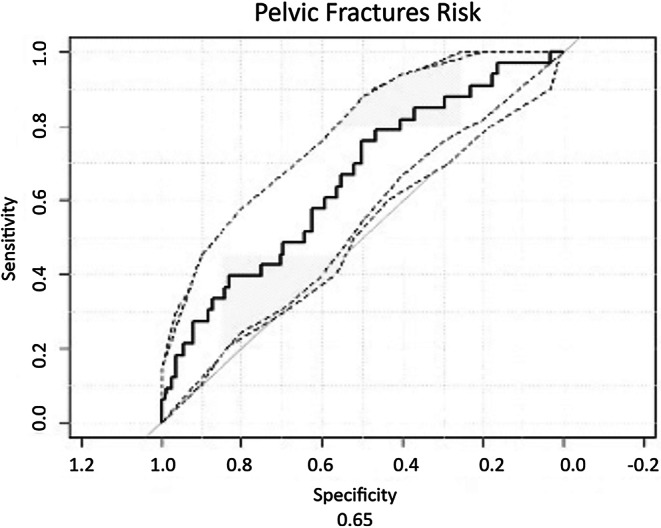
Receiver operating characteristic curve of risk factors for the development of post-radiotherapy pelvic fractures

Univariate analysis revealed that both age (*p* = 0.003) and V40 of the LPBM (*p* = 0.04) are statistically significant risk factors for the development of pelvic fractures after RT. Combining the two parameters, a logistic regression model was obtained with an AUC of 0.67 (95% confidence interval: 0.55–0.77), a sensitivity of 83.3%, and a specificity of 61.5% (Fig. 1).

**Fig. 2:**
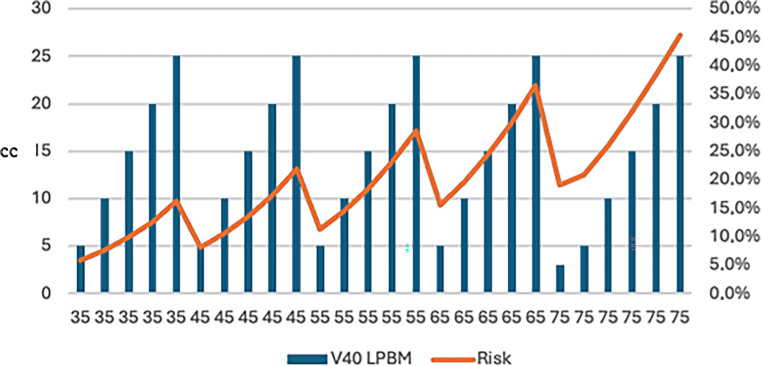
Fracture risk (right y axis) as a function of age (x axis) and V40 of the lower pelvis bone marrow (*LPBM*; left y axis)

Considering the two variables included in the predictive model, the probability of post-RT pelvic fracture occurrence was defined across varying age categories and dose constraints related to V40 of the LPBM (Fig. 2).

## Discussion

Today, pelvic RT is considered an essential treatment for cervical cancer. The recent optimization of RT treatment, the use of new irradiation techniques (intensity-modulated radiation therapy [IMRT] or VMAT), and the introduction of concomitant platin-based chemotherapy have led to a significant increase in patients survival rates. Consequently, increasing attention has been paid to the potential late effects on the pelvic region resulting from RT treatment. In fact, PIF is an important topic, given the increasing survival rates of patients and the potential impact of PIFs on quality of life. However, these fractures remain underreported in the literature and are often underappreciated in clinical practice.

This study shows that PIFs represent a relatively common late toxicity occurring in patients receiving definitive chemoradiation therapy for locally advanced cervical carcinoma. In our series, 36 of 202 patients (16.7%) developed at least one fracture after definitive RT with VMAT. The majority of PIFs were located in the sacrum (81%), in agreement with previous studies that described the sacrum as the most frequently implicated bone for pelvic fractures after RT [12]. Likewise, Ramlov et al. [14] underlined that the sacrum was the most common site of fracture (77%) and that all patients developed at least one sacral fracture. Consistent with other meta-analyses, only a limited number of patients reported pelvic or lumbar symptoms, accounting for 33% of women diagnosed with PIF [16, 17].

Our study was based on a retrospective evaluation of patients’ morbidity and clinical characteristics, as well as a systematic review of repeated MRI scans, and included both symptomatic and asymptomatic PIFs. With this strategy, we aimed to identify all potential pathological events for a more complete account of the effects of irradiation on pelvic bones. Moreover, the study was designed to analyze a group of patients treated uniformly for locally advanced cervical cancer who received both EBRT and ICBT.

The median time interval between the end of RT and the detection of PIF through MRI was 7 months in our analysis, which is in line with findings from previous studies (6–16.9 months). These data are supported by the Bazire et al. [6] and Ramlov et al. [14] studies, which reported that most PIFs occurred within 1 year after the end of EBRT.

Pelvic insufficiency fractures occur as a result of normal physiological stress on bones previously weakened by demineralization and reduced elastic resistance. As a consequence, any factor that promotes changes in the bone matrix may contribute to the development of the disease. Certainly, osteoporosis represents the leading risk factor for bone insufficiency fractures [18]. Postmenopausal state and age have been reported as the most relevant risk factors for post-RT PIF in several studies [6, 18, 19], consistent with the finding that the most important risk factor is osteoporosis. Low CT density of bone and BM, smoking, BMI, long-term corticosteroid therapy, and other conditions have all been evaluated as potential contributing factors in the occurrence of PIF in different meta-analyses [7, 15, 20, 21]. In accordance with previous findings, the median age of patients was associated with a high prevalence of PIF and emerged as the most predictive clinical parameter for the development of pelvic fractures in our series. An association between the occurrence of PIFs and other clinical features (such as smoking, BMI, CT density) was not observed in this study, even though they are known risk factors for osteoporosis [22]. The difficulties related to the correlation analysis may reflect the limitations of this study, a well-known problem in retrospective studies. Almost all patients received cisplatin. Concomitant chemotherapy is therefore not a variable that can be tested in regard to PIF risk.

According to the KEYNOTE-18 trial, the addition of pembrolizumab to standard chemoradiotherapy has redefined the treatment paradigm for advanced cervical cancer. In our cohort, which is comparable to the trial population, we did not observe an increased incidence of PIFs despite the use of similar RT target volumes and dose constraints. No additional skeletal toxicity emerged with the integration of immunotherapy.

Despite the recent growing interest in the literature regarding post-RT PIFs, definitive and certain information on their incidence and contributing factors is still lacking. In particular, there are only few clinical studies investigating the potential association between dosimetric parameters and the risk of developing these complications after RT treatment. To date, no dosimetric study has defined a specific dose constraint that must be observed to considerably lower the risk of PIF occurrence.

Therefore, this study represents one of the first reports aiming to identify a potential association between dosimetric parameters and the occurrence of pelvic fractures. Previous studies have suggested the benefit of IMRT in sparing bone and consequently decreasing bone toxicity [23]. Nevertheless, this benefit has not been completely demonstrated. Thus, we performed a BM-sparing procedure as described by Mell et al. [10], with the primary intention of saving the BM, reducing the dose to the pelvic bones, and optimizing the dose to the sacrum. Our data showed that the dose of IMRT delivered to lower spinal and sacral BM represents a significant predisposing factor for the development of pelvic fractures. Risk probability rates of the occurrence of PIFs were then calculated in relation to the two variables included in the aforementioned predictive model, age and the dose related to V40of the LPBM. Furthermore, we proposed possible dose constraints for V40 of the LPBM according to patient age to reduce the probability of developing post-RT pelvic fractures.

However, we would like to emphasize that the majority of fractures in our study were located in the sacrum, whereas the dosimetric parameter that emerged as predictive was related to the LPBM. This finding may be explained by the beam rearrangement adopted in our treatment approach, as previously reported in the literature, which results in proportionally higher dose exposure to the sacrum within the LPBM volume. In a future prospective study that also accounts for the numerous clinical factors known to influence fracture risk, additional dosimetric predictors specifically related to the lumbosacral bone marrow may be identified.

### Limitations

This study has several limitations. First, it is a retrospective study conducted at a single institution. The limited data available and the small number of patients enrolled in this study may contribute to limitations in statistical interpretation. Moreover, the interval and protocol of imaging studies were not standardized, leading to potential detection bias. Post-treatment MRIs were primarily aimed at evaluating the response to therapy rather than seeking for bone fractures in the irradiated fields, which may have led to an underestimation of fractures in the upper pelvis or spine. Furthermore, the follow-up period was neither uniform nor sufficiently long to accurately estimate the true incidence of fractures in this population.

## Conclusion

This analysis provides an estimation of the incidence of pelvic insufficiency fractures (PIF) in patients with locally advanced cervical cancer treated with a combination of intensity-modulated radiation therapy and chemotherapy. Clinical and dosimetric parameters were evaluated as potential risk factors in the development of pelvic fractures. In this study, an age-dependent dose constraint was proposed to reduce the risk of PIF after radiotherapy treatment: This constraint focused on optimizing the 40 Gy isodose to the lumbosacral pelvis. However, further studies should aim to validate our results by incorporating these parameters into the planning process. Moreover, future active interventions, such as early counseling and surveillance, should be included in the oncological follow-up of patients with cervical cancer who undergo radiotherapy.
